# A Theacrine-Based Supplement Increases Cellular NAD^+^ Levels and Affects Biomarkers Related to Sirtuin Activity in C2C12 Muscle Cells In Vitro

**DOI:** 10.3390/nu12123727

**Published:** 2020-12-03

**Authors:** Petey W. Mumford, Shelby C. Osburn, Carlton D. Fox, Joshua S. Godwin, Michael D. Roberts

**Affiliations:** 1 School of Health Sciences, Lindenwood University, Saint Charles, MO 63301, USA; pmumford@lindenwood.edu (P.W.M.); cdf0007@auburn.edu (C.D.F.); jsg0061@auburn.edu (J.S.G.); 2 School of Kinesiology, Auburn University, Auburn, AL 36849, USA; sco0004@auburn.edu

**Keywords:** theacrine, mRNAs, sirtuins, mitochondria

## Abstract

There is evidence in rodents to suggest that theacrine-based supplements modulate tissue sirtuin activity as well as other biological processes associated with aging. Herein, we examined if a theacrine-based supplement (termed NAD3) altered sirtuin activity in vitro while also affecting markers of mitochondrial biogenesis. The murine C2C12 myoblast cell line was used for experimentation. Following 7 days of differentiation, myotubes were treated with 0.45 mg/mL of NAD3 (containing ~2 mM theacrine) for 3 and 24 h (*n* = 6 treatment wells per time point). Relative to control (CTL)-treated cells, NAD3 treatments increased (*p* < 0.05) Sirt1 mRNA levels at 3 h, as well as global sirtuin activity at 3 and 24 h. Follow-up experiments comparing 24 h NAD3 or CTL treatments indicated that NAD3 increased nicotinamide phosphoribosyltransferase (NAMPT) and SIRT1 protein levels (*p* < 0.05). Cellular nicotinamide adenine dinucleotide (NAD^+^) levels were also elevated nearly two-fold after 24 h of NAD3 versus CTL treatments (*p* < 0.001). Markers of mitochondrial biogenesis were minimally affected. Although these data are limited to select biomarkers in vitro, these preliminary findings suggest that a theacrine-based supplement can modulate select biomarkers related to NAD^+^ biogenesis and sirtuin activity. However, these changes did not drive increases in mitochondrial biogenesis. While promising, these data are limited to a rodent cell line and human muscle biopsy studies are needed to validate and elucidate the significance of these findings.

## 1. Introduction

Skeletal muscle aging is associated with numerous factors including (but not limited to) age-associated alterations in circulating hormones, low-grade inflammation, mitochondrial perturbations via free radicals, and transcriptomic alterations due to epigenetic factors (reviewed in [1,2,3]). There are various nutritional supplements geared towards affecting biomarkers associated with tissue aging in general, and several of these supplements act through reducing inflammation (e.g., curcumin, [4]), diminishing oxidative stress (e.g., vitamins C and E, [5]), and enhancing mitochondrial function (e.g., coQ10 and quercetin, [6,7]).

Sirtuins are a class of proteins (SIRT1-7) that exist in various subcellular compartments and possess either mono-ADP-ribosyltransferase or deacetylase activity [8]. Research dating back to the 1970s across several organismal models have indicated that sirtuins are involved in the tissue aging process; specifically, stressors that activate sirtuin activity are largely thought to promulgate anti-aging effects [9]. Thus, there has also been a widespread interest in identifying nutritional supplements that increase cellular sirtuin activity and/or nicotinamide adenine dinucleotide (NAD^+^) levels given that NAD^+^ increases sirtuin activity [10,11].

Theacrine is a purine alkaloid that is structurally similar to caffeine, and various studies have examined its efficacy as a neuroactive ingredient [12,13,14]. Aside from potential neurotrophic effects, there are limited data in rodents suggesting theacrine-based supplements can reduce inflammation [15,16], modulate mitochondrial function [17], and activate sirtuins [17]. However, it is currently unclear how theacrine-based supplements affect the biomarkers related to these processes in skeletal muscle. Therefore, the purpose of this rapid report was to assess how a theacrine-based supplement (NAD3) affected markers of sirtuin activity and mitochondrial biogenesis skeletal muscle cells in vitro.

## 2. Methods

### 2.1. Cell Culture

Cell culture methods were performed similarly to other studies published by our laboratory [18,19]. Briefly, C2C12 myoblasts (passage 6; American Type Culture Collection, Manassas, VA, USA), were seeded in Dulbecco’s Modified Eagle Media (DMEM) with 10% FBS, 1% penicillin/streptomycin, and 0.1% gentamycin ((Corning Inc., Corning, NY, USA) on six-well plates at a seeding density of 3 × 10^5^ under standard culture conditions (37 °C in a 5% CO_2_ atmosphere). Once myoblast growth reached 80–90% confluence ~48 h after seeding, differentiation was induced by removing the growth medium and replacing it with a differentiation medium (DM; DMEM, 2% (*v*/*v*) horse serum, 1% penicillin/streptomycin, and 0.1% gentamycin). DM was then replaced every 24 h for 7 days to allow for adequate myotube growth.

### 2.2. NAD3 Versus Control Treatments

Treatments occurred on day 7 post-differentiation for either 3 or 24 h with either 0.45 mg/mL of NAD3 (containing ~2 mM theacrine; Compound Solutions, Carlsbad, CA, USA) (*n* = 6 treatment wells per time point) or a weight-equivalent of cellulose-only (*n* = 6 treatment wells per time point) suspended in DM. Notably, we opted not to resuspend the treatments in a vehicle prior to adding these agents to DM (e.g., ethanol or dimethylsulfoxide) given the potential cytotoxic effects of vehicle compounds. Rather, NAD3 or cellulose were added to DM in 50 mL tubes, tubes were centrifuged for 5 min at 100× *g* to remove any insoluble material, and the supernatant was used as the treatment media. Notably, the investigators were blinded to treatments until the conclusion of the study and after statistical analyses were performed.

### 2.3. Post-Treatment Processing of Cells and Real-Time PCR for mRNA Expression Analyses

After all treatments, the differentiation/treatment media were removed, and the cells were washed once with phosphate-buffered saline (PBS). Thereafter, the PBS was siphoned off and then a subset of cells were scraped from a plate and transferred into 250 µL of Trizol for RNA isolation. Following Trizol-based RNA isolation methods, total RNA concentrations were analyzed using a Nanodrop Lite spectrophotometer (Thermo Fisher Scientific, Waltham, MS, USA), and 1 µg of cDNA was synthesized using a commercial qScript cDNA SuperMix (Quanta Biosciences, Gaithersburg, MD, USA) per the manufacturer’s recommendations. Real-time PCR was performed using gene-specific primers and SYBR green chemistry, and all PCR reactions were confirmed to produce only one melt product. Primer sequences can be found in the Supplemental Data. Relative expression values were performed using the 2^−ΔΔCT^ method where 2^−ΔCT^ (housekeeping gene CT—gene of interest CT) and 2^−ΔΔCT^ (or fold change) = (2^−ΔCT^ value/2^−ΔCT^ average of control treatment). Cyclophilin (*Ppia*) was used as a housekeeping gene.

After the RNA scrape described above, 250 µL of ice-cold cell lysis buffer was applied to each well (20 mM Tris·HCl (pH 7.5), 150 mM NaCl, 1 mM Na-EDTA, 1 mM ethylene glycol-bis(β-aminoethyl ether)-N,N,N′,N′-tetraacetic acid, 1% Triton, 20 mM sodium pyrophosphate, 25 mM sodium fluoride, 1 mM β-glycerophosphate, 1 mM Na3VO4, and 1 µg/mL leupeptin; Cell Signaling; Danvers, MA, USA). Plates were then scraped, and the cell slurry was removed and placed in 1.7 mL tubes. Cells were homogenized via micropestles, and homogenates were subsequently centrifuged at 500× *g* for 5 min. After centrifugation, insoluble proteins were pelleted, and the supernatants containing solubilized cell material were placed in new 1.7 mL tubes and stored at −80 °C until the global sirtuin (SIRT) activity and citrate synthase assays were performed as described below.

### 2.4. Global SIRT Activity and Citrate Synthase Activity Assays

Global SIRT activity levels were performed on cell lysis buffer lysates using a commercial assay (Abcam, Danvers, MA, USA; catalog#: Ab156915) similar to previous methods published by our laboratory [20]. Prior to performing the assay, protein concentrations of supernatants were determined using a BCA assay (Thermo Fisher Scientific). Lysates (8 μL) were loaded in duplicate onto 96-well plates provided by the kit for enzymatic reactions as well as a no SIRT co-factor control reaction (also called an NNC reaction, or an internal negative control reaction). Following the execution of the assay as per the manufacturer’s recommendations, absorbances were read at OD450 using a plate reader (BioTek Synergy H1). NNC optical density (OD) values were subtracted from enzymatic reaction OD values, and these values are presented in the results section as OD450 per μg protein. The average coefficient of variation (CV) values for all duplicates was 10.3%.

Citrate synthase activity levels were determined in duplicate on lysates from 24 h treatments only; notably, these methods are similar to previous methods used by our laboratory [20]. This metric was used as a surrogate for mitochondrial density as per the findings of Larsen et al. [21], suggesting that citrate synthase activity strongly correlates with transmission electron micrograph images of intracellular space occupied by mitochondria (*r* = 0.84, *p* < 0.001). The assay principle is based on the reduction of 5,50-dithiobis (2-nitrobenzoic acid) (DTNB) at 412 nm (extinction coefficient 13.6 mmol/L/cm) coupled to the reduction of acetyl-CoA by the citrate synthase reaction in the presence of oxaloacetate. Briefly, protein obtained from lysates was added to a mixture composed of 0.125 mol/L Tris–HCl (pH 8.0), 0.03 mmol/L acetyl-CoA, and 0.1 mmol/L 5,5′-dithiobis-(2-nitrobenzoic acid). All reactions occurred in 96-well plates, reactions were initiated by the addition of 5 μL of 50 mmol/L oxaloacetate per well, and the absorbance change was recorded for 60 s in a spectrophotometer (BioTek Synergy H1). The average CV values for all duplicates was less than 10%.

### 2.5. Follow-Up Culture Experiments for Western Blotting Targets and Determination of NAD^+^ Concentrations

To validate some of our qPCR targets identified above, we performed follow-up experiments in myotubes derived from C2C12 myoblasts 7 days post-differentiation (ATCC, passage 2) (Table S1). Due to limited resources, we only performed NAD3 or control (CTL) treatments for 24 h. The assayed protein targets for these experiments included SIRT1/6 protein levels (given the *Sirt1* mRNA alterations) as well as PGC1-α and NRF2 protein levels (as markers of mitochondrial biogenesis). We also opted to interrogate nicotinamide phosphoribosyltransferase (NAMPT) protein levels, given its role in NAD^+^ synthesis.

Western blotting was performed in an identical fashion to previous studies performed in our laboratory. For experimental details, readers should refer to [20,22]. Notably, densitometry values for each target were quantified using a gel documentation system and imaging software (Image Lab; BioRad, Hercules, CA, USA). Likewise, Ponceau images were also obtained to ensure the equal loading of proteins, and the density of each target of interest was divided by Ponceau density to obtain a relative expression value. The primary antibodies chosen for the molecular targets were as follows: SIRT1, rabbit IgG (Cell Signaling, Danvers, MA, USA; cat# 9475); SIRT6, rabbit IgG (Cell Signaling; cat# 3868); NAMPT, rabbit IgG (Abcam, Cambridge, MA, USA; catalog#: Ab45890); NRF2, rabbit IgG (Novus Biologicals; catalog#: NBP1-32822); PGC1-α, rabbit IgG (Novus Biologicals; catalog#: NBP1-04676B).

Cells treated with NAD3 or CTL for 24 h were also assayed for cellular NAD^+^, NADH, and NADt (NAD^+^ + NADH) concentrations using an enzymatic kit (Abcam; cat# ab65348) and methods previously described by our laboratory [20]. Raw absorbance values provided for each variable were normalized to the average of the control condition and expressed as relative concentrations.

### 2.6. Statistics

All statistical analyses were performed using SPSS v25.0 (IBM Corp, Armonk, NY, USA). The 3 h and 24 h data were analyzed independently between treatments using independent samples t-tests. All data are presented in figures and tables as the means ± standard deviation (SD) values, and statistical significance was established as *p* < 0.050.

## 3. Results

### 3.1. Effect of NAD3 Treatments on SIRT Markers

**Figure 1:**
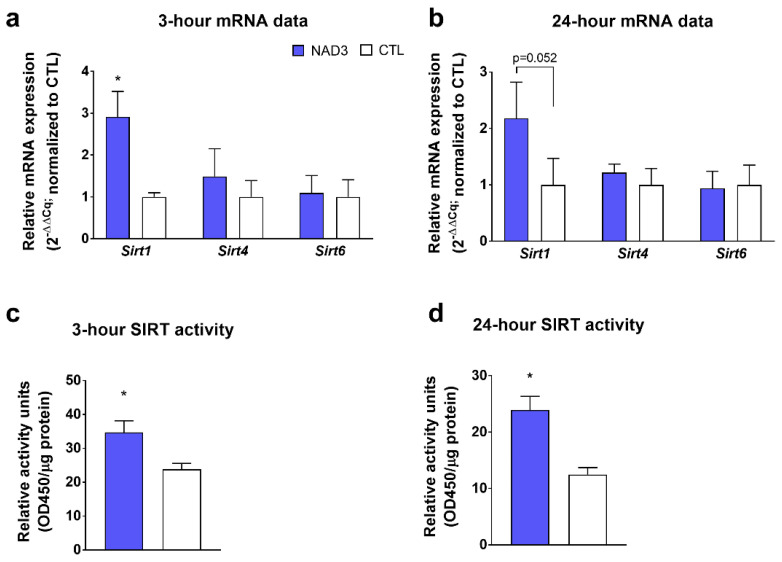
Effects of experimental (NAD3) treatments on sirtuin (SIRT) markers. These data illustrate 3 h treatments on SIRT-related mRNAs (panel **a**), 24 h treatments on SIRT-related mRNAs (panel **b**), 3 h treatments on global SIRT activity (panel **c**), and 24 h treatments on global SIRT activity (panel **d**). All data are presented as the means ± standard deviation values (*n* = 6 treatment wells per condition), and significance was set at *p* < 0.05. Symbol: *, indicates more highly expressed in NAD3-treated versus control (CTL)-treated cells. Other abbreviation: OD, optical density.

Data in Figure 1 illustrate that 3 h NAD3 treatments increased the mRNA expression of *Sirt1* relative to CTL-treated cells (*p* < 0.001), while not affecting *Sirt4* or *Sirt6* mRNA expression levels (Figure 1a,b). Additionally, 3 h and 24 h NAD3 treatments increased global SIRT activity (*p* = 0.018 and *p* = 0.002, respectively; Figure 1c,d).

### 3.2. Effect of NAD3 Treatments on Mitochondrial Biogenesis Markers

**Figure 2:**
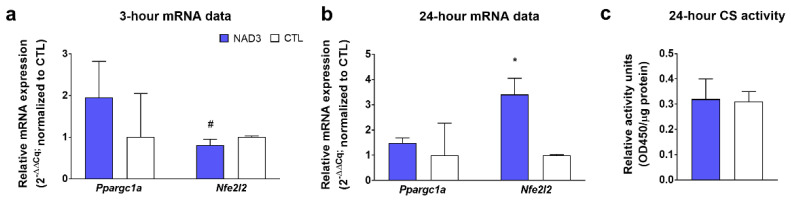
Effects of NAD3 treatments on mitochondrial biogenesis markers. Legend: these data illustrate 3 h treatments on mRNAs related to mitochondrial biogenesis (panel **a**), 24 h treatments on mRNAs related to mitochondrial biogenesis (panel **b**), and 24 h treatments on a surrogate marker of mitochondrial volume (panel **c**). All data are presented as the means ± standard deviation values (*n* = 6 treatment wells per condition), and significance was set at *p* < 0.05. Symbols: *, indicates more highly expressed in NAD3-treated versus control (CTL)-treated cells; #, indicates more highly expressed in control (CTL)-treated versus NAD3-treated cells. Other abbreviation: CS, citrate synthase.

Data in Figure 2 illustrate that, while 3 and 24 h NAD3 treatments did not affect *Ppargc1a* mRNA levels, 3 h NAD3 treatments decreased (*p* = 0.010) and 24 h NAD3 treatments increased (*p* < 0.001) *Nfe2l2* mRNA levels relative to CTL-treated cells (*p* < 0.05) (Figure 2b). However, 24 h NAD3 treatments did not affect citrate synthase activity levels (Figure 2c).

### 3.3. Effects of 24-Hour NAD3 Treatments on Protein Targets

**Figure 3:**
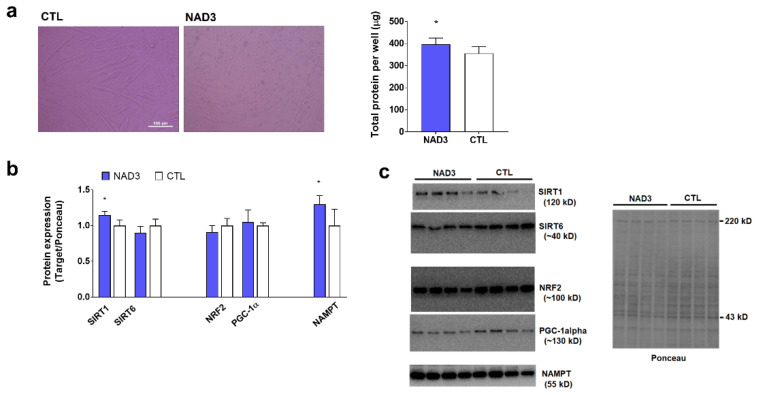
Effects of NAD3 treatments on select protein targets. Legend: these data illustrate 24 h NAD3 treatments that do not affect cellular morphology, while also increasing protein levels per well (a surrogate of cellular health) (panel **a**). Twenty-four hour NAD3 treatments also increased SIRT1 and NAMPT protein levels, while not affecting other protein targets (panel **b**). Panel **c** also illustrates representative Western blots of each assayed target. All data are presented as the means ± standard deviation values (*n* = 5–6 treatment wells per condition), and significance was set at *p* < 0.05. Symbols: *, indicates more highly expressed in NAD3-treated versus control (CTL)-treated cells.

Cellular morphology was not altered with 24 h NAD3 treatments, and the total protein per well (a surrogate of cell health) was surprisingly increased with 24 h NAD3 versus control treatments (*p* = 0.035) (Figure 3a). Additionally, 24 h NAD3 treatments increased SIRT1 and NAMPT protein levels (*p* = 0.003 and *p* = 0.024, respectively), while not affecting the protein levels of SIRT6 (*p* = 0.097), PGC-1α (*p* = 0.484), or NRF2 (*p* = 0.109) (Figure 3b).

### 3.4. Effect of 24-hour NAD3 Treatments on Cellular NAD^+^, NADH, and NAD^+^/NADH

**Figure 4:**
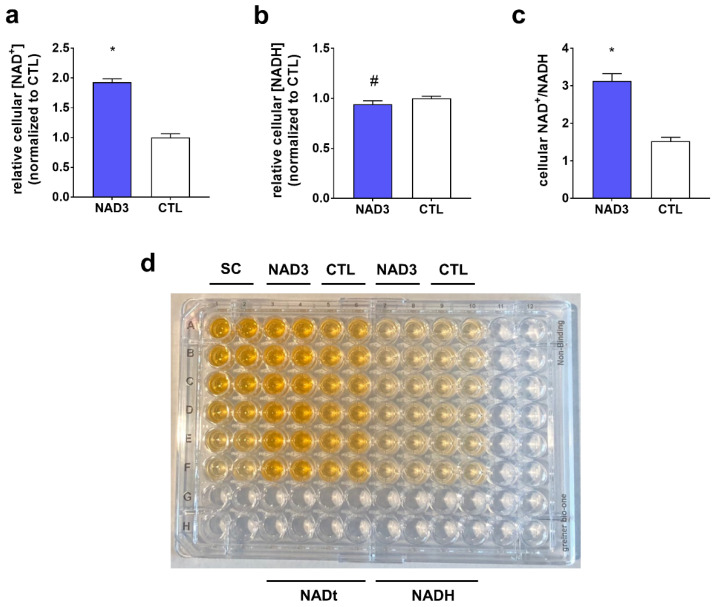
Effect of 24 h NAD3 treatments on cellular NAD^+^, NADH, and NAD^+^/NADH. Legend: these data illustrate that 24 h NAD3 treatments increased cellular NAD^+^ levels (panel **a**), decreased NADH levels (panel **b**), and increased the NAD^+^/NADH ratio (panel **c**). (Panel **d**) illustrates a visual representation of assay results (SC indicates a standard curve, and NADt is NAD^+^ + NADH where NAD^+^ levels are determined by subtracting NADH levels from NADt levels). All data are presented as the means ± standard deviation values (*n* = 6 treatment wells per condition), and significance was set at *p* < 0.05. Symbols: *, indicates more highly expressed in NAD3-treated versus control (CTL)-treated cells; #, indicates more highly expressed in CTL-treated versus NAD3-treated cells.

We sought to determine how 24 h NAD3 treatments affected given that: (a) global sirtuin activity was robustly increased by 3 h and 24 h NAD3 treatments; (b) NAD^+^ activates sirtuins as previously discussed; and (c) 24 h NAD3 treatments increased NAMPT protein levels, this being an enzyme involved in NAD^+^ biosynthesis as discussed later. Data in Figure 4 illustrate that 24 h NAD3 treatments increased cellular NAD^+^ concentrations by nearly two-fold (*p* < 0.001; Figure 4a). Interestingly, NAD3 also caused a slight (~6%), but significant downregulation in cellular NADH concentrations (Figure 4b). Finally, the NAD^+^/NADH ratio was elevated nearly two-fold in NAD3-treated versus CTL-treated cells (*p* < 0.001; Figure 4c).

## 4. Discussion

This is the first study to examine how NAD3, a theacrine-based supplement, affects molecular markers in skeletal muscle cells. Notably, NAD3 treatments increased *Sirt1* mRNA levels as well as global sirtuin activity relative to CTL-treated cells. Follow-up experiments on 24 h treated cells indicated NAD3 treatments increased SIRT1 protein levels, NAMPT protein levels, and cellular NAD^+^ concentrations. However, while certain mRNA markers related to mitochondrial biogenesis were altered with NAD3 treatments, citrate synthase activity levels (a surrogate of mitochondrial volume) were not affected with 24 h NAD3 treatments. These findings are discussed in greater detail below.

As mentioned previously, there is widespread interest in augmenting cellular sirtuin activity and NAD^+^ concentrations given that decrements in sirtuin activity as well as NAD^+^ levels have been implicated in tissue aging [20]. The NAD3-induced increase in global sirtuin activity and Sirt1 mRNA levels agrees, in part, with prior literature. For instance, Wang et al. [17] determined that theacrine activates SIRT3 activity in liver cells in vitro. Song et al. [23] similarly reported that theacrine increases SIRT3 protein levels in myocardial tissue. Due to cell lysate constraints, we opted to measure global sirtuin activity rather than the individual activities of various sirtuins. Likewise, resource constraints prevented the interrogation of mRNA and protein levels of all sirtuins. However, it is certainly possible that NAD3 preferentially activated the activity of individual sirtuins leading to the observed increases in global sirtuin activity herein. Moreover, our data showing that NAD3 treatments upregulate the mRNA and protein levels of SIRT1 suggest that enhanced global sirtuin activity levels may occur through an increase in this specific sirtuin. The NAD3-induced increase in NAMPT protein levels is interesting given that NAD+ biosynthesis can be catalyzed through the salvage/recycling pathway and NAMPT is the rate-limiting enzyme in this pathway [24]. The two-fold increase in cellular NAD^+^ concentrations after 24 h of NAD3 treatment may be related to the aforementioned effect and critically, this too may be a mechanism that is involved with the robust increase in sirtuin activity observed herein. Collectively, while our findings are limited to an unperturbed cell culture model, these findings are promising given the role that sirtuins have in skeletal muscle; specifically, their role in enhancing mitochondrial function and the transcriptional control of various metabolic genes [20].

In spite of the NAD3-induced increase in global sirtuin activity as well as the NAD3-induced increase in *Nfe2l2* mRNA levels, protein levels of NRF2 as well as mitochondrial biogenesis (as assessed through citrate synthase activity) remained unaltered. Although this finding is difficult to reconcile, it may be possible that NAD3, rather than appreciably affecting mitochondrial biogenesis, enhanced mitochondrial function. While we did not assess metrics of mitochondrial function (e.g., oxidation rates), it is notable that Wang et al. [17] reported that theacrine enhanced mitochondrial fat oxidation in liver cells. Moreover, the authors noted that these effects were likely mediated through theacrine-induced increases in SIRT3 activity given that this enzyme acts to deacetylate various mitochondrial protein in order to increase their activity. Thus, in light of these findings, future research is needed to determine if NAD3 enhanced mitochondrial function in skeletal muscle.

### Experimental Considerations

There are limitations to the current data. First, these data are in vitro, and muscle biopsy studies in humans are needed to confirm and refine the current findings. Another limitation to these data is that only select biomarkers were interrogated in one cell type. Thus, more studies are needed to extensively assess the molecular alterations induced by NAD3 treatments in human cell lines as well as multiple cell types. Moreover, while there are seven sirtuin isotypes, qPCR was performed on only three of these isotypes due to RNA yield limitations, and follow-up Western blotting was performed on SIRT1/6 due to resource constraints. Notwithstanding, these preliminary findings regarding the effects of a theacrine-based supplement global sirtuin activity, NAMPT protein levels, and cellular NAD^+^ concentrations are promising and warrant future research in vivo.

## 5. Conclusions

Although these data are in vitro, this preliminary evidence suggests a theacrine-based supplement can increase SIRT activity, NAMPT protein levels, and cellular NAD^+^ concentrations. However, and as mentioned prior, human studies are needed to validate the current findings.

## Supplementary Materials

The following are available online at https://www.mdpi.com/2072-6643/12/12/3727/s1, Table S1: Primer sequences used for qPCR.
