# Supplementary material for: A Theacrine-Based Supplement Increases Cellular NAD+ Levels and Affects Biomarkers Related to Sirtuin Activity in C2C12 Muscle Cells In Vitro
Source: Nutrients. 2020 Dec 3;12(12):3727. doi: 10.3390/nu12123727 (PMC7761648; doi:10.3390/nu12123727)
Supplement: Supplementary file 1 [file nutrients-12-03727-s001.pdf]

Gene	Forward primer (5′ → 3′)	Reverse primer (5′ → 3′)
Sirt1	AGTTCCAGCCGTCTCTGTGT	CTCCACGAACAGCTTCACAA
Sirt4	TCCAAAGGCTGGAAATGAAC	GCGACACAGCTACTCCATCA
Sirt6	GGGAACTTGAAGGAACCACA	AGCCTGGGCTATAGCAGTGA
Ppargc1a	TCGCAGAAGCAGTGTTCCAT	CCATGGTCGTATCAGAGGCC
Nfe2l2	CTCGCTGGAAAAAGAAGTGG	CCGTCCAGGAGTTCAGAGAG
Ppia	GTGGTCTTTGGGAAGGTGAA	TTACAGGACATTGCGAGCAG

Supplemental data. Primer sequences used for qPCR.
